# GSVD- and tensor GSVD-uncovered patterns of DNA copy-number alterations predict adenocarcinomas survival in general and in response to platinum

**DOI:** 10.1063/1.5099268

**Published:** 2019-08-20

**Authors:** Matthew W. Bradley, Katherine A. Aiello, Sri Priya Ponnapalli, Heidi A. Hanson, Orly Alter

**Affiliations:** 1 Scientific Computing and Imaging Institute, University of Utah, Salt Lake City, Utah 84112, USA; 2 Department of Bioengineering, University of Utah, Salt Lake City, Utah 84112, USA; 3 Utah Population Database and Department of Surgery, University of Utah, Salt Lake City, Utah 84112, USA; 4 Huntsman Cancer Institute, University of Utah, Salt Lake City, Utah 84112, USA; 5 Department of Human Genetics, University of Utah, Salt Lake City, Utah 84112, USA

## Abstract

More than a quarter of lung, uterine, and ovarian adenocarcinoma (LUAD, USEC, and OV) tumors are resistant to platinum drugs. Only recently and only in OV, patterns of copy-number alterations that predict survival in response to platinum were discovered, and only by using the tensor GSVD to compare Agilent microarray platform-matched profiles of patient-matched normal and primary tumor DNA. Here, we use the GSVD to compare whole-genome sequencing (WGS) and Affymetrix microarray profiles of patient-matched normal and primary LUAD, USEC, and OV tumor DNA. First, the GSVD uncovers patterns similar to one Agilent OV pattern, where a loss of most of the chromosome arm 6p combined with a gain of 12p encode for transformation. Like the Agilent OV pattern, the WGS LUAD and Affymetrix LUAD, USEC, and OV patterns are correlated with shorter survival, in general and in response to platinum. Like the tensor GSVD, the GSVD separates these tumor-exclusive genotypes from experimental inconsistencies. Second, by identifying the shorter survival phenotypes among the WGS- and Affymetrix-profiled tumors, the Agilent pattern proves to be a technology-independent predictor of survival, independent also of the best other indicator at diagnosis, i.e., stage. Third, like no other indicator, the pattern predicts the overall survival of OV patients experiencing progression-free survival, in general and in response to platinum. We conclude that comparative spectral decompositions, such as the GSVD and tensor GSVD, underlie a mathematically universal description of the relationships between a primary tumor's genotype and a patient's overall survival phenotype, which other methods miss.

## INTRODUCTION

LUAD, USEC, and OV tumors account for ≳12%, ≲0.5%, and ≳2% of cancer deaths in the US, respectively. More than a quarter of the primary tumors are resistant to platinum-based chemotherapy, i.e., the first-line systemic treatment to accompany surgery for over three decades.^1–3^ Most primary tumors are followed by new tumor events, e.g., metastasis, recurrence, or progression, even in patients experiencing complete remission and months of progression-free survival (PFS) from the end of the treatment of the primary tumor. Over successive new tumor events, most tumors develop resistance to platinum, defined by PFS shorter than six months. Yet, no indicator exists which predicts—past the end of the primary treatment and during, e.g., the first PFS interval—the benefit of platinum in terms of overall survival of LUAD, USEC, and adenocarcinomas in general.

LUAD, USEC, and OV tumor cells are thought to derive selective advantages from a prevalence of DNA copy-number alterations (CNAs) rather than from recurrent point mutations.^4,5^ Even in the normal human genome, copy-number variations (CNVs) are 10^2^–10^4^ times more frequent than point mutations.^6,7^ Yet, despite advances in genomic profiling technologies and the growing number of publicly available genomic data, e.g., in the Cancer Genome Atlas (TCGA)^8–10^ recurrent CNAs were not identified in adenocarcinomas that were translated into clinical use.

Only recently and only in OV, patterns of DNA CNAs were discovered which predict the overall survival of patients, in general as well as following the platinum-based treatment of the primary tumor and throughout the course of the disease.^11,12^ The patterns, across the chromosome arms 7p and, separately, Xq and across the combination of the two arms 6p + 12p together but not separately, were discovered by using the tensor GSVD, a “comparative spectral decomposition,” to compare Agilent microarray platform-matched profiles of patient-matched normal and primary OV tumor DNA. Like other profiling technologies, these Agilent comparative genomic hybridization (CGH) microarrays rely on a specific experimental design and a specialized computational protocol, which is sensitive to perturbations to the data, e.g., due to the changes in the experimental batch or the computational preprocessing. This has contributed to a low reproducibility, <70% between technical replicates of the same sample and <50% between computational assessments of the same raw data, in assigning CNVs in normal DNA or CNAs in tumor DNA.^13^

Here, we show that one of these genotype-phenotype relations is appropriate for adenocarcinomas other than OV, e.g., USEC, which like OV is a gynecological disease, and LUAD, which is the most common form of lung cancer, the leading cause of cancer death in the US and worldwide among both women and men (Methods and Fig. S1 in the supplementary material and Datasets S1–S4). We also show that these relations are suitable for profiling technologies other than Agilent CGH microarrays, i.e., whole-genome sequencing (WGS),^14^ and Affymetrix single nucleotide polymorphism (SNP) microarrays, which together with the Agilent CGH microarrays represent the main technologies.

## COMPARATIVE SPECTRAL DECOMPOSITIONS

We formulated the GSVD and defined a tensor GSVD to be a comparative spectral decomposition, i.e., to simultaneously identify the similarities and dissimilarities between two column-matched but row-independent matrices and tensors, respectively, and thus create a single coherent model from two datasets recording different aspects of interrelated phenomena.^15–18^ Any two datasets that record patient-matched tumor and normal genomes or, more than that, technology-matched profiles of patient-matched tumor and normal genomes are of such matrix or tensor structures, respectively. The column axes correspond to the patients and the genomic profiling technologies, respectively, and are shared. The row axes correspond to the regions covered by the microarray probes or WGS bins across the tumor and, separately, normal genomes, and are independent.

The unsupervised, data-driven GSVD and tensor GSVD separate the two datasets, of shared column axes but independent row axes, into pairs of combinations of patterns. Each pair includes one combination of patterns from each dataset, where the patterns across the shared column axes are identical, but the patterns across the independent row axes are, in general, different between the two combinations. The two sets of patterns across the two independent row axes are uncorrelated, i.e., orthogonal. Each pair of combinations of patterns corresponds to a pair of generalized singular values or “tensor generalized singular values,” respectively, which are the superposition coefficients of the first combination in the first dataset and the second combination in the second dataset. When the second coefficient is negligible, i.e., insignificant, relative to the first, the first combination of patterns is interpreted to represent the phenomena exclusive to the first dataset. When the first coefficient in one pair is large in magnitude, i.e., significance, among all the first coefficients in all pairs, the corresponding first combination of patterns is interpreted to represent the phenomena that are significant to the first dataset.

In a comparison of patient-matched tumor and normal genomes, therefore, the GSVD can uncover the unique combinations of patterns of variations across the patients and the tumor and normal genomic regions, which mathematically are significant in and exclusive to the tumor genomes. In a comparison of technology-matched profiles of patient-matched tumor and normal genomes, the tensor GSVD can uncover the combinations of patterns that mathematically additionally are consistent across the technologies. Biologically and medically, such combinations of patterns can be expected to describe disease mechanisms and predict patient outcomes, respectively.

### The GSVD formulated as a comparative spectral decomposition

A mathematical building block of computational algorithms and physical theories, the GSVD of the two matrices $D_{i}\in R^{K_{i}\times L}$, both with full column rank *L* ≤ *K_i_*, exists and simultaneously factorizes both *D_i_* into two sets of basis vectors each, a *D_i_*-specific column-wise orthonormal, i.e., orthogonal and normalized, $U_{i}\in R^{K_{i}\times L}$ and a shared invertible row-normalized $V^{T}\in R^{L\times L}$,^19,20^

$$D_{i}=U_{i}\Sigma_{i}V^{T}=\sum_{a=1}^{L}\sigma_{i,a}(u_{i,a}⊗v_{a}^{T}),\;i=1,2.$$ (1)

The positive diagonal core matrices $\Sigma_{i}=\text{diag}(\sigma_{i,l})\in R^{L\times L}$ list the generalized singular values in a decreasing order of the ratio *σ*_1,__*a*_/*σ*_2,__*a*_. The GSVD is unique up to the phase factors of ±1 of each triplet of basis vectors *u_i_*_,__*a*_ and *v_a_*, except in degenerate subspaces defined by the subsets of equal *σ*_1,__*a*_/*σ*_2,__*a*_.

**FIG. 1.**
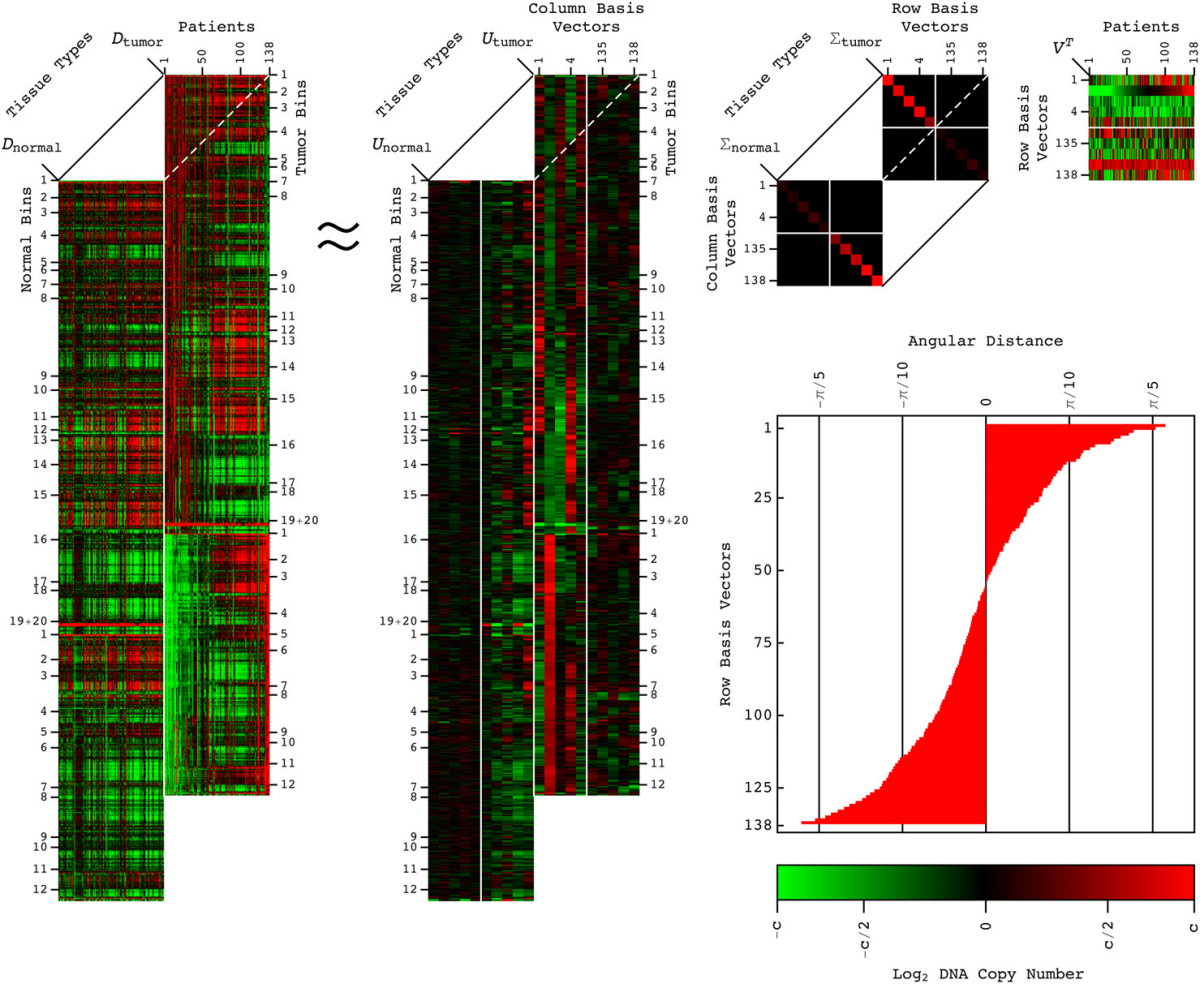
The GSVD of the 6p + 12p WGS profiles of patient-matched LUAD tumor and normal DNA. The GSVD of Eq. (1) is depicted in a raster display with a relative WGS read-count, i.e., DNA copy-number amplification (red), no change (black), and deletion (green). This GSVD depiction is denoted as approximate, even though the GSVD is exact, because only the first through the 5th and the 134th through the 138th row and the corresponding tumor and normal column basis vectors and generalized singular values are explicitly shown. The angular distances of Eq. (2) are depicted in the bar chart in the inset. The red and green contrasts for the datasets *D_i_*, the dataset-specific column basis vectors *U_i_* and generalized singular values Σ_*i*_, and the shared row basis vectors *V^T^* are *c *=* *1, 150 and 7.5 × 10^−4^, and 10, respectively.

To formulate the GSVD as a comparative spectral decomposition, we defined the “GSVD angular distance,” i.e., the significance of the combination of *u*_1,__*a*_ and *v_a_* in *D*_1_ relative to that of *u*_2,__*a*_ and *v_a_* in *D*_2_, to be a function of *σ*_1,__*a*_/*σ*_2,__*a*_ that, from the cosine-sine decomposition, is related to an angle (Figs. 1 and S2)

$$-\pi /4<\theta_{a}=\arctan(\sigma_{1,a}/\sigma_{2,a})-\pi /4<\pi /4.$$ (2)

A unique combination of *u*_1,__*a*_ and *v_a_* with a GSVD angular distance of *θ_a_* ≈ *π*/4, i.e., a ratio of *σ*_1,__*a*_/*σ*_2,__*a*_ ≫ 1, is, therefore, mathematically approximately exclusive to *D*_1_, and for consistency should be interpreted to represent the phenomena exclusive to the first dataset.

### The tensor GSVD is a comparative spectral decomposition

To extend the GSVD, we defined a tensor GSVD of two, e.g., third-order, tensors $D_{i}\in R^{K_{i}\times L\times M}$, which with *LM* ≤ *K_i_* unfold into three pairs of full column-rank matrices *D_i_*, *D_ix_*, and *D_iy_*. The tensor GSVD simultaneously factorizes both $D_{i}$ into three sets of basis vectors each, a $D_{i}$-specific column-wise orthonormal $U_{i}\in R^{K_{i}\times \mathrm{LM}}$ and shared invertible row-normalized $V_{x}^{T}\in R^{L\times L}$ and $V_{y}^{T}\in {R,}^{M\times M}$

$$\begin{array}{c} D_{i}=R_{i}\times_{a}U_{i}\times_{b}V_{x}\times_{c}V_{y}\\ =\sum_{a=1}^{\mathrm{LM}}\sum_{b=1}^{L}\sum_{c=1}^{M}r_{i,\mathrm{abc}}(u_{i,a}⊗v_{x,b}^{T}⊗v_{y,c}^{T}), \end{array}$$ (3)

where ×_*a*_*U*_*i*_, ×_*b*_*V*_*x*_, and ×_*c*_*V*_*y*_ denote multiplications of the matrices with the core tensor $R_{i}\in R^{\mathrm{LM}\times L\times M}$ and ⊗ denotes an outer product.

The basis vectors of the tensor GSVD are computed from the GSVDs of the tensors unfolded into three pairs of full column-rank matrices

$$\begin{array}{c} U_{i}\Sigma_{i}V^{T}=D_{i}=[\cdots ,D_{i,:\mathrm{lm}},\ldots ]\in R^{K_{i}\times \mathrm{LM}},\\ V_{x}\Sigma_{\mathrm{ix}}U_{\mathrm{ix}}^{T}=D_{\mathrm{ix}}^{T}=[\cdots ,D_{i,k:m},\ldots ]\in R^{L\times MK_{i}},\\ V_{y}\Sigma_{\mathrm{iy}}U_{\mathrm{iy}}^{T}=D_{\mathrm{iy}}^{T}=[\cdots ,D_{i,\mathrm{kl}:},\ldots ]\in R^{M\times K_{i}L}, \end{array}$$ (4)

where $D_{i,:\mathrm{lm}}$ is the vector in $D_{i}$, which spans the *K_i_*-row dimension, in the *l*th position along the *L*-*x*-column dimension and the *m*th position along the *M*-*y*-column dimension. The full core tensors $R_{i}$ are computed by contracting $D_{i}$ with $U_{i}^{T},\;V_{x}^{-T}$, and $V_{y}^{-T}$, e.g., by unfolding the core tensors into the matrices

$$\begin{array}{c} R_{i}=[\cdots ,R_{i,:\mathrm{bc}},\ldots ]\in R^{\mathrm{LM}\times \mathrm{LM}},\\ R_{i}=U_{i}^{T}D_{i}(V_{y}^{-T}⊗V_{x}^{-T})=\Sigma_{i}V^{T}(V_{y}^{-T}⊗V_{x}^{-T}), \end{array}$$ (5)

where $U_{i}^{T}D_{i}=\Sigma_{i}V^{T}$ from the GSVD of *D_i_* and ⊗ denotes a Kronecker product. The ratios of the tensor generalized singular values *r_i_*_,__*abc*_, therefore, equal the ratios of the corresponding row mode generalized singular values *σ_i_*_,__*a*_, i.e.,

$$\begin{array}{c} |r_{1,\mathrm{abc}}/r_{2,\mathrm{abc}}|=r_{1,\mathrm{abc}}/r_{2,\mathrm{abc}}=r_{1,a}/r_{2,a}\\ =\sigma_{1,a}/\sigma_{2,a}>0. \end{array}$$ (6)

It follows that the “tensor GSVD angular distance,” i.e., the significance of the basis vectors *u*_1,__*a*_, *v_x_*_,__*b*_, and *v_y_*_,__*c*_ in $D_{1}$ relative to that of *u*_2,__*a*_, *v_x_*_,__*b*_, and *v_y_*_,__*c*_ in $D_{2}$, equals the row mode GSVD angular distance

$$\Theta_{\mathrm{abc}}=\arctan(|r_{1,\mathrm{abc}}/r_{2,\mathrm{abc}}|)-\pi /4=\theta_{a}.$$ (7)

A unique combination of basis vectors *u*_1,__*a*_, *v_x_*_,__*b*_, and *v_y_*_,__*c*_ with a tensor GSVD angular distance of Θ_*abc*_ = *θ_a_* ≈ *π*/4, therefore, is mathematically approximately exclusive to $D_{1}$ and for consistency should be interpreted in terms of the interrelations among the different aspects of the phenomena which are exclusive to the first dataset. This interpretation of the tensor GSVD as a comparative spectral decomposition is possible because, like the GSVD, the tensor GSVD is exact, exists, and has unique properties that directly generalize those of the SVD^21,22^ (Theorem S1).

## AN ADENOCARCINOMA GENOTYPE-PHENOTYPE RELATION

Consider, e.g., one combination of basis vectors previously uncovered by a tensor GSVD comparison of patient-matched normal and primary OV tumor profiles across the two chromosome arms 6p + 12p. The profiles were measured twice, by a set of two Agilent CGH microarray platforms. Mathematically, the combination is unique and is the most significant in the tumor genomes, i.e., corresponds to the largest tensor generalized singular value in the tumor dataset. It includes the column basis vector across the tumor set of probes which mathematically is the most exclusive to the tumor relative to the normal genomes, i.e., corresponds to the largest ratio of tensor generalized singular values in the tumor relative to the normal dataset. Biologically, the Agilent OV pattern that corresponds to this vector describes co-occurring DNA CNAs that encode for transformation. These include most of the CNAs that were known and several that were unrecognized in OV prior to the discovery of the pattern. Medically, the pattern predicts the overall survival by identifying the patients of a shorter survival phenotype in the Agilent OV discovery and, separately, also validation sets, of 249 and 148 patients, respectively, indicating that the survival phenotype is related to the genotype. Similarly, the *x*-row basis vector across the discovery set of patients identifies a subset of patients of statistically significantly shorter survival times. The *y*-row basis vector across the set of platforms is approximately constant, indicating that this genotype-phenotype relation is independent of the Agilent microarray platform.

**FIG. 2.**
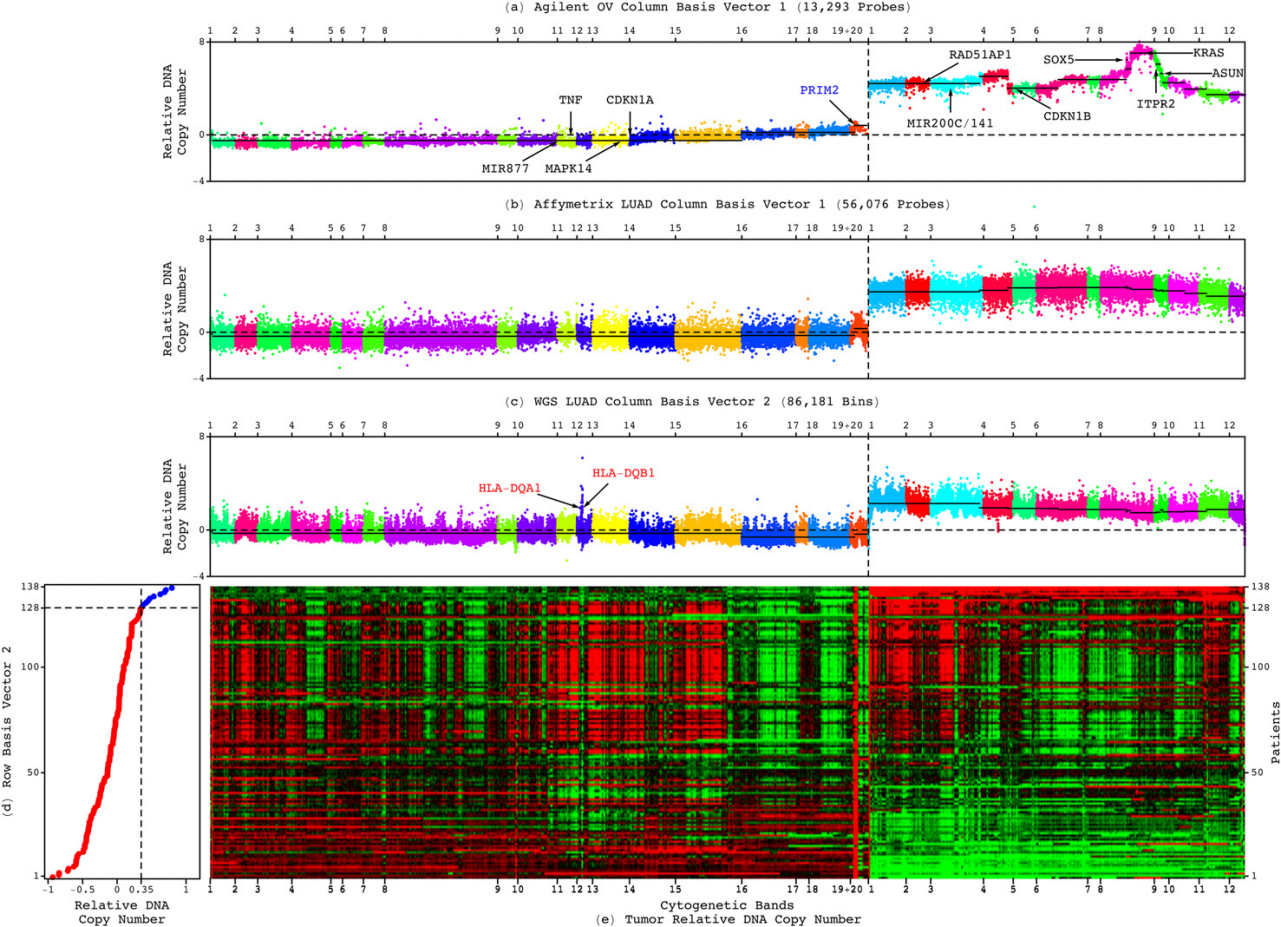
An adenocarcinoma genotype-phenotype relation. The (a) Agilent OV, (b) Affymetrix LUAD, and (c) WGS LUAD patterns, which correspond to column basis vectors that are significant in and exclusive to the tumor genomes, are depicted in plots of relative copy numbers, ordered and colored based upon genomic coordinates, with the medians of the segments identified in the Agilent OV pattern by CBS (black lines), including OV-specific (blue), adenocarcinoma-shared (black), and WGS technology-filled in, possibly LUAD-specific (red) CNAs. (d) The corresponding WGS LUAD row basis vector is depicted in the plot showing the classification of the 138 patients into low (red) or high (blue) superposition coefficients. (e) The WGS LUAD tumor dataset is depicted in a raster showing the genotype-phenotype relation.

Here, we find that the GSVD comparisons of patient-matched normal and primary LUAD, USEC, and OV tumor profiles measured by Affymetrix SNP microarrays and normal and primary LUAD tumor profiles measured by WGS uncover similar combinations across 6p + 12p. Mathematically, these combinations are unique and are significant in and exclusive to the tumor genomes (Fig. S3). Biologically, the WGS LUAD and Affymetrix LUAD, USEC, and OV patterns that correspond to the column basis vectors in these combinations are similar to the Agilent OV pattern (Figs. 2 and S4) and describe LUAD and USEC genotypes that are similar to the OV genotype. Medically, the corresponding row basis vectors identify the subsets of patients of shorter LUAD, USEC, and OV survival phenotypes.

### Genotypes of a loss of most of 6p combined with a gain of 12p encode for transformation

To compare the genotypes, we classified the genomic segments previously identified in the Agilent OV pattern by using circular binary segmentation (CBS)^23^ as amplified, unaltered, or deleted in the WGS LUAD and Affymetrix LUAD, USEC, and OV patterns (Dataset S5). The classification is based upon the differences, in the median absolute deviation (MAD), between the copy-number medians of the segments and the chromosome arms 6p + 12p. Of the 14 segments of >30 Agilent probes, 12 are classified the same in the five adenocarcinoma patterns. These describe an adenocarcinoma-shared genotype of a loss of a segment of ≈46M nucleotides, i.e., >75% of 6p, and a gain of 12p.

Of the two remaining segments, one describes a LUAD-specific loss of an additional segment of >11M nucleotides, i.e., 18% of 6p. The second describes an OV-specific amplification at the centromeric end of 6p. By filling in gaps in the genome which are not covered by the microarray probes, the WGS LUAD pattern adds an amplification of 33 K nucleotides within the 46M-nucleotide deletion on 6p, which may be LUAD specific.

**FIG. 3.**
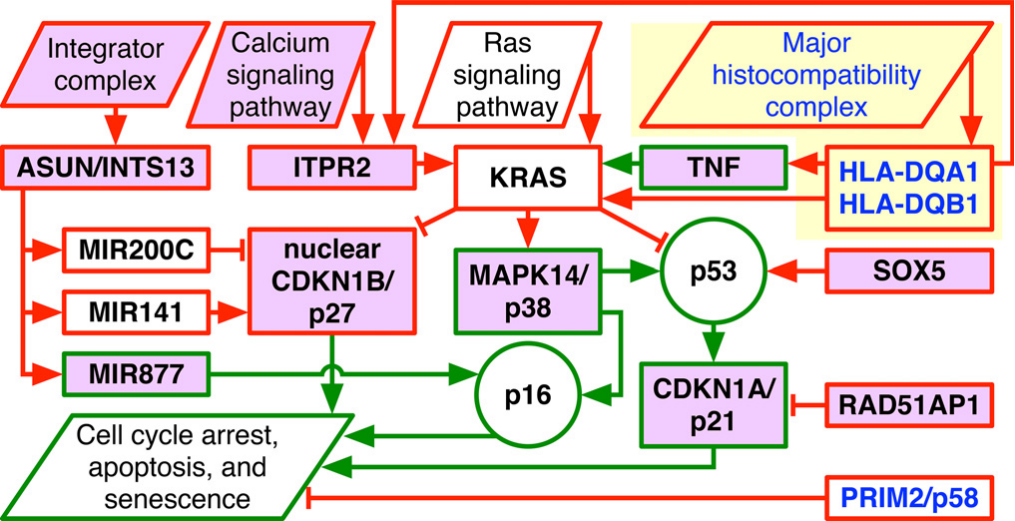
The adenocarcinoma genotypes encode for transformation via the Ras pathway supported by the calcium pathway and the integrator complex. The 6p + 12p LUAD, USEC, and OV genotypes are depicted in a diagram of the WGS technology-filled in, possibly LUAD-specific, multihistocompatibility complex (yellow) in addition to the Agilent microarray-described Ras and calcium pathways and integrator complex, which include CNAs unrecognized in adenocarcinomas prior to the discovery of the Agilent OV pattern (violet). Explicitly shown are amplifications (red) and deletions (green) of genes and transcript variants (rectangles), either adenocarcinoma-shared (black) or specific (blue), and relationships that directly or indirectly lead to increased (arrows) or decreased (bars) activities of the genes and transcripts and the tumor suppressor proteins p53 and p16 (circles).

We find the adenocarcinoma-shared deletions of the mitogen-activated protein kinase p38-encoding *MAPK14* and the cyclin-dependent kinase (CDK) inhibitor p21-encoding *CDKN1A* on 6p and amplifications of the Rad51 associated protein-encoding *RAD51AP1* and the Kirsten rat sarcoma viral oncogene homolog-encoding *KRAS* on the 12p encode—together but not separately—for transformation via the Ras signaling pathway (Fig. 3). These naturally occurring alterations are analogous to the genetic elements that artificially convert human normal cells into tumor cells.^24^ While it is still an open question how these alterations occur,^25^ the absence of CDK inhibitors together with the presence of DNA damage was shown to lead to cells with polyploid nuclei.^26,27^

Additional adenocarcinoma-shared CNAs involve the calcium signaling pathway and the integrator complex in support of Ras-mediated transformation. The amplification of the inositol 1,4,5-trisphosphate receptor-encoding *ITPR2* on 12p can activate Ras by releasing calcium ions from the endoplasmic reticulum.^28^ The amplification of *ASUN/INTS13*, a subunit of the integrator complex, which is essential for the 3′-end processing of small nuclear RNAs, can enhance the expression of microRNAs (miRNAs), e.g., miR-200c and miR-141 on 12p,^29,30^ and regulate the mRNA expression in a length-dependent manner in tumor vs normal cells.^31,32^ The oncogenic herpesvirus saimiri, e.g., expresses its viral miRNAs by using the integrator complex of its host.^33^

We also find that CNAs that are specific to any one of the adenocarcinomas additionally promote transformation. The WGS technology filled-in, possibly LUAD-specific, amplified segment on 6p encompasses the major histocompatibility complex (MHC) class II human leukocyte antigen (HLA) alpha and beta chain-encoding *HLA-DQA1* and *HLA-DQB1*. Upon detecting an antigen, MHC II induces the Ras and calcium pathways by binding to the T-lymphocyte cell receptor complex.^34^ MHC II ligands also activate the Ras pathway by stimulating the expression of the tumor necrosis factor-encoding *TNF*.^35,36^ Note that *HLA-DQA1* and *HLA-DQB1* are highly expressed in Epstein-Barr virus-transformed B-lymphocytes, where they mediate the viral entry.^37^ The OV-specific amplified segment on 6p includes the 3′-end of the largest transcript variant of the gene *PRIM2* that encodes the human DNA primase large subunit, i.e., p58, and is essential for DNA replication initiation. The amplification starts at the seventh intron of *PRIM2*, which is also the second intron of the conserved eukaryotic and archaeal primase domain within *PRIM2*. Insertions of murine leukemia viruses (MLVs) that do not contain oncogenes into different sites within the same intron were observed to induce tumorigenesis in mice.^38^ The OV-specific amplification, like the MLV insertions, may lead to overexpression of the 3′-end of *PRIM2* and most of the conserved primase subunit and, therefore, support transformation. Note that *PRIM2* is overexpressed in mouse models of human epithelial breast, lung, and prostate cancers that are induced by the expression of the oncoproteins of simian virus 40, i.e., the large and small tumor antigens.^39^

### Phenotypes of shorter survival, in general and in response to platinum

Like the 6p + 12p Agilent OV pattern, the WGS LUAD and Affymetrix LUAD, USEC, and OV patterns predict the overall survival by identifying subsets of patients of shorter survival phenotypes, which are of median survival times of roughly one and a half years in LUAD and USEC and three years in OV (Tables S1–S4). Of the 470 LUAD patients with matched Affymetrix tumor and normal profiles, e.g., 36 are classified as having high weights of the Affymetrix LUAD pattern in their tumor profiles based upon the superposition coefficients listed in the row basis vector that corresponds to the pattern. Of the same 470 patients, 34, including 30 of the 36, i.e., ≈88%, have high Spearman correlations of their tumor profiles with the pattern. Similarly, of the 140 platinum-treated patients among the 470, 14 have high correlations with the pattern, including 13, i.e., ≈93% of 14 that have high coefficients. We use the correlation cutoff of ≈0.35 and compute the coefficient cutoff by scaling 0.35 by the Frobenius norm of the vector that lists the correlations, as was previously established and validated for the Agilent OV discovery and validation sets of patients, respectively.

In Kaplan-Meier (KM) survival analyses of the set of 470 LUAD patients and, separately, the subset of 140 platinum-treated patients, the subsets of patients with high superposition coefficients and, separately, Spearman correlations, are of a median survival time of an approximately one and a half years, statistically significantly shorter than that of the complement subsets of patients. In Cox proportional hazards models, a high coefficient or, separately, correlation, confers, in general, ≳2.3 times the hazard and, in response to platinum, ≳3.5 times the hazard of a low coefficient or correlation, respectively.

Note that the sizes of the subsets of LUAD, USEC, and OV patients of shorter survival differ from the complement subsets. The subset of 36 of the 470 LUAD patients, e.g., corresponds to ≳7.5% of the patients. The 14 of the 140 platinum-treated patients correspond to 10%. However, while the KM analyses and Cox models reflect the subset sizes, these analyses and models do not assume subsets and complement subsets of equal sizes.

### Blind separation from experimental sources of the copy-number variation

Both the GSVD and tensor GSVD separate the tumor and normal datasets into combinations of patterns across the tumor and normal genomic regions, which are uncorrelated, i.e., orthogonal, and patterns across the patients, which, in general, are normalized. Only the tensor GSVD additionally separates the datasets into patterns across the platforms that can be constant, i.e., consistent across the profiling technologies. We find, however, that like the tensor GSVD, the GSVD blindly, i.e., in an unsupervised, data-driven manner, separates the adenocarcinoma genotype-phenotype relation from technology-specific experimental variations that affect the minimally preprocessed WGS, Affymetrix, and Agilent profiles. These include the WGS-specific effects of the guanine-cytosine (GC) content variations across the tumor and normal genomes. The magnitude of these effects varies between Illumina sequencers.

The first WGS LUAD tumor and 138th normal column basis vectors, which are the most exclusive to the WGS LUAD tumor and normal profiles, respectively, are also the most significant in the tumor and normal profiles, and are correlated with the fractional GC content across the tumor and normal genomes, respectively, with both correlations ≳0.72 and both Mann-Whitney-Wilcoxon (MWW) *P*-values $<10^{-10^{3}}$ (Figs. S5–S7). Both vectors roughly describe the frequent spikes of increased copy numbers superimposed on an invariant baseline in agreement with the polymerase chain reaction (PCR) amplification-dependent WGS technology underestimating the abundance of GC-poor sequences.^40^ The corresponding first and 138th row basis vectors are correlated with experimental variations in the Illumina sequencers of the tumor and normal DNA with both hypergeometric and MWW *P*-values <10^−2^ (Fig. S8). The GSVD mathematically identifies the variations in the sequences to be exclusive to the tumor and, separately, normal profiles, because the sequencers are unmatched between the patient-matched tumor and normal DNA.

## A PREDICTOR OF ADENOCARCINOMAS SURVIVAL INDEPENDENT OF THE BEST OTHER INDICATOR AT DIAGNOSIS, i.e., THE TUMOR STAGE

**FIG. 4.**
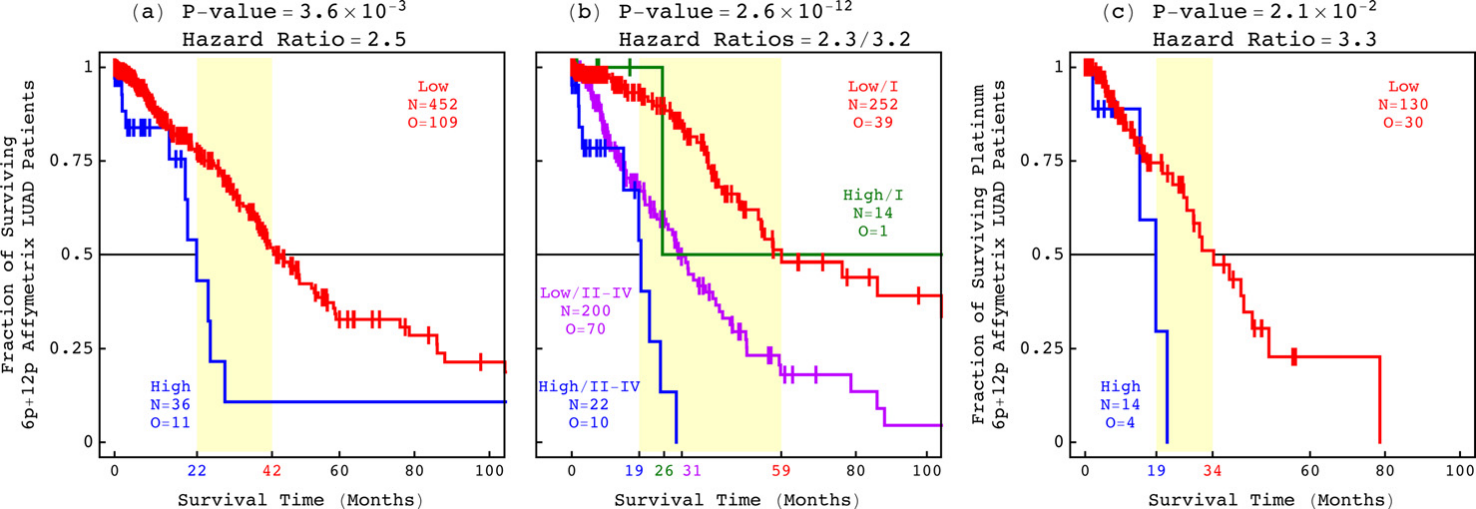
The 6p + 12p Agilent OV pattern is a technology-independent predictor of LUAD overall survival, independent also of the best other indicator at diagnosis, i.e., stage. The classifications of (a) the 488 Affymetrix LUAD patients based upon the Agilent OV pattern and, in addition, (b) stage and of (c) the 144 platinum-treated patients among the 488 based upon the Agilent OV pattern are depicted in KM curves, highlighting the median survival time differences (yellow) with the corresponding log-rank *P*-values and Cox hazard ratios.

To compare the phenotypes, we additionally classified the adenocarcinoma patients based upon the correlations of the 6p + 12p Agilent OV pattern with the WGS LUAD and Affymetrix LUAD, USEC, and OV tumor profiles. We find that the pattern predicts the survival of the LUAD and USEC, as well as the OV patients, in general and in response to platinum, and independent of the profiling technology (Figs. 4, S9, and S10). Of the 488 LUAD patients with Affymetrix tumor profiles, e.g., 36 have high correlations with the pattern, including 31, i.e., ≈91% of 34 that have high correlations with the Affymetrix LUAD pattern. Similarly, of the 144 platinum-treated patients among the 488, 14 have high correlations with the Agilent OV pattern, including 13, i.e., ≈93% of 14 that have high correlations with the Affymetrix LUAD pattern. In KM analyses of the 488 and, separately, 144 patients, the subsets of patients identified by the Agilent OV and, separately, Affymetrix LUAD patterns are statistically indistinguishable, with the corresponding log-rank *P*-values >0.05. In Cox models of the 488 patients, the classifications based upon the patterns are statistically consistent, where the corresponding hazard ratios of ≈2.5 and 2.3 are within the 95% confidence interval of each other, and the concordance indices, i.e., accuracies, of ≈76% and 77% are similar.

The Agilent OV pattern is a predictor independent of the best other indicator at diagnosis, i.e., the tumor stage. In the Cox models of the 488 LUAD patients, e.g., the bivariate hazard ratios of the Agilent OV and, separately, Affymetrix LUAD patterns and stage are within the 95% confidence intervals of the corresponding univariate ratios, and the bivariate concordance indices are similar to the univariate ones.

The Agilent OV pattern is independent of intratumor heterogeneity as it is reflected in the TCGA parameters of the tumor sample's volume, the slide's percent tumor cells and nuclei, the portion's weight, and the analyte's and aliquot's DNA concentrations, with the corresponding MWW *P*-values >0.05. The effect of intratumor heterogeneity as it may be reflected in DNA extracted from different portions of the primary tumor is limited to only two, i.e., <1.5%, of the 139 LUAD patients with both Affymetrix and WGS profiles, and 21, i.e., <6.5%, of the 331 OV patients with both Agilent and Affymetrix profiles, having classifications that are inconsistent between the profiles.

The Agilent OV pattern, of co-occurring CNAs, is consistent with the differential expression of genes and miRNAs (Figs. S11–S13). Of the 15 genes and miRNAs highlighted in the genotypes, 11 are overexpressed or underexpressed in at least one of the subsets of adenocarcinoma tumors that have high correlations with the Agilent OV pattern, with the corresponding MWW *P*-values <0.05. These subsets of tumors correspond to the subsets of patients that have shorter survival phenotypes. Of these 11 genes and miRNAs, 10, i.e., ≈90%, consistently map to amplifications or deletions in the WGS LUAD and Affymetrix LUAD, USEC, and OV patterns.

## THE PRIMARY TUMOR'S GENOTYPE PREDICTS THE PATIENT'S OVERALL SURVIVAL PHENOTYPE THROUGHOUT THE DISEASE

The leading causes of death from adenocarcinomas in general, and LUAD, USEC, and OV in particular, are new tumor events, e.g., metastasis, recurrence, or progression. Most patients experience some PFS following the end of the primary treatment. Yet, among the indicators of adenocarcinomas in clinical use, including the tumor stage at diagnosis, the residual disease after surgery, and the therapy outcome and neoplasm status after chemotherapy, none predicts the overall survival of patients experiencing PFS. Similarly, no indicator predicts—past the end of the primary treatment and during the first PFS interval—the benefit of platinum-based chemotherapy as a first-line systemic treatment in terms of overall survival.

**FIG. 5.**
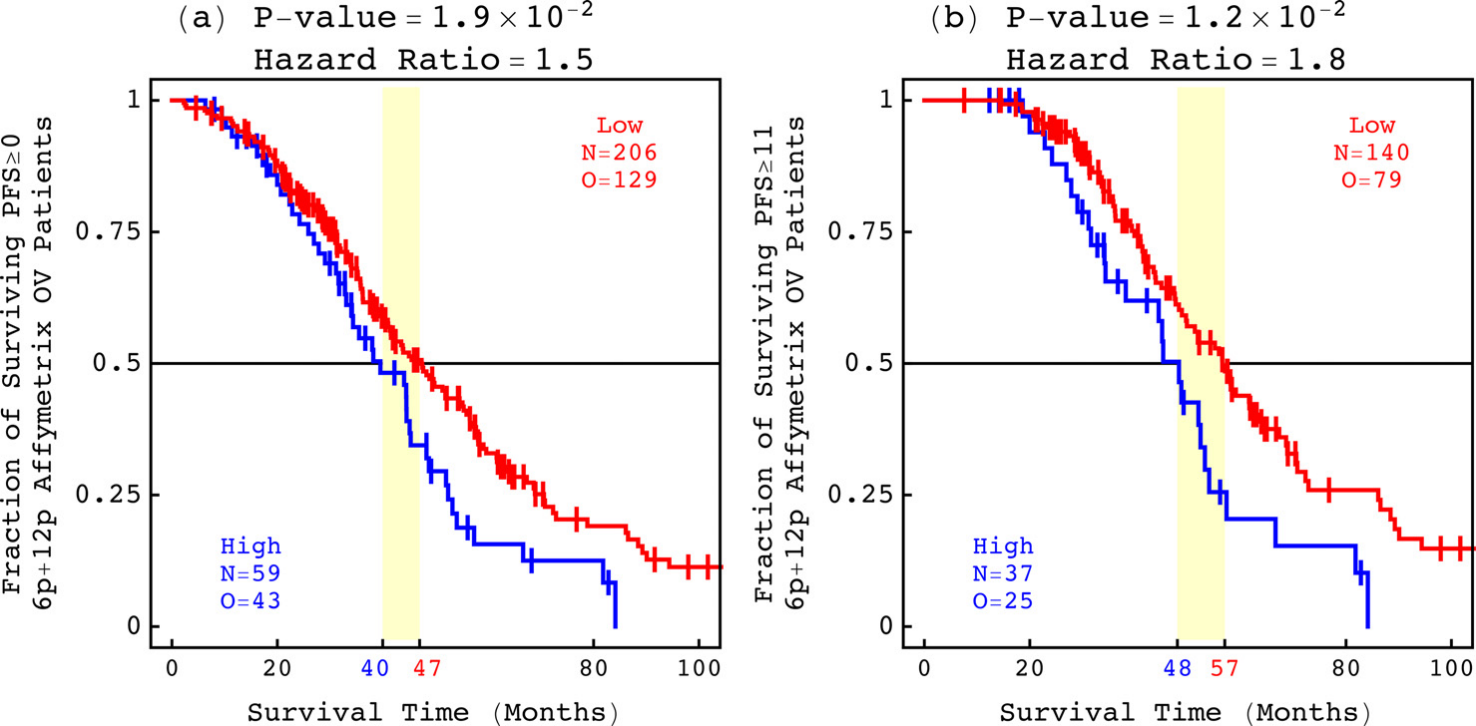
The 6p + 12p Agilent OV pattern predicts the overall survival of OV patients experiencing PFS. The classifications of (a) 265 of the 479 Affymetrix OV patients who experienced PFS ≥ 0 months and (b) 177 of the 265 patients who experienced PFS ≥ 11 months, based upon the 6p + 12p Agilent OV pattern.

We find that, like no other indicator, the 6p + 12p Agilent OV pattern is a technology-independent predictor of the overall survival of patients experiencing PFS, in general and in response to platinum, which may be OV specific (Fig. 5). Additional, OV-specific, predictors are the 7p and Xq Agilent OV patterns (Fig. S14 and Tables S5–S7).

Consider, e.g., the KM analyses of the set of 479 OV patients, the subset of 265 of the 479 OV patients who experienced PFS ≥ 0 months, and the subset of 177 of the 265 patients who experienced PFS ≥ 11 months. The median survival time of the shorter survival phenotype identified by the Agilent OV pattern increases from roughly three years for the 479 and 265 patients to four years for the 177 patients. The KM median survival time of the shorter survival phenotype, therefore, correctly reflects the PFS period experienced by the patients. However, the hazard ratios and concordance indices of the corresponding Cox models remain statistically indistinguishable. The corresponding hazard ratios of ≈1.6, 1.5, and 1.8 for the 479, 265, and 177 patients are within the 95% confidence intervals of each other, and the concordance indices of ≈56%, 58%, and 64% are similar. A high correlation with the Agilent OV pattern, therefore, confers the same hazard relative to a low correlation, regardless of the PFS period.

## DISCUSSION

That the adenocarcinoma genotype-phenotype relation holds past the end of the primary treatment, in general and in response to platinum, implies that the information contained in the primary tumor's genome, even though it may be affected by the primary tumor's treatment, is relevant to the mechanisms of adenocarcinomas throughout the disease.

That the genotype-phenotype relation is statistically independent of the best other indicator of adenocarcinomas at diagnosis, i.e., the primary tumor's stage, implies that the information contained in the relation is not currently being used in clinical practice. This information includes, e.g., drug targets and combinations of drug targets that are predicted to be correlated with a patient's outcome. Note that there already exist drugs, some of which are FDA approved but not necessarily for any one of the adenocarcinomas, that interact with, e.g., *MAPK14*, *CDKN1A*, and *RAD51AP1*.^41^ By using this information in clinical practice, therefore, it can be expected to improve the prognostics, diagnostics, and therapeutics of the disease.

That the adenocarcinoma genotype-phenotype relation is invariably uncovered by, and only by, the GSVD and tensor GSVD, independent of the adenocarcinoma type and the profiling technology, highlights the role of mathematics in genomic data science and machine learning. As comparative spectral decompositions, the GSVD and tensor GSVD simultaneously identify the similarities and dissimilarities between the primary tumor and patient-matched normal genomes. These unsupervised, data-driven decompositions use the structure of the datasets, of two column-matched but row-independent matrices and tensors, respectively, in the blind source separation (BSS) of the tumor-exclusive genotypes and phenotypes from experimental batch effects. This makes the decompositions sensitive to robust genotype-phenotype relations in small discovery sets of as few as 138 WGS LUAD, 109 Affymetrix USEC, and 249 Agilent OV patients and possibly imbalanced validation sets of, e.g., 148 Agilent OV patients, with large genomic profiles of, e.g., >86 K WGS bins, 51 K Affymetrix, and 13 K Agilent probes, respectively.

Only recently, a copy-number genotype predictive of a brain astrocytoma survival phenotype was discovered only by using the GSVD.^42^ In comparisons of astrocytoma tumor and patient-matched normal genomes, the GSVD invariably separated the tumor-exclusive genotype and phenotype from those that occur in the normal genomes and from experimental batch effects, independent of the astrocytoma type and the profiling technology. The tumor-exclusive genotype invariably predicted the survival phenotype statistically better than any other indicator of astrocytoma. For decades prior to this discovery, recurring DNA alterations have been observed in astrocytoma without being translated into clinical use because attempts to associate the tumor copy-number genotypes with patient outcome phenotypes were unsuccessful.^43–45^

We conclude that by using the complex structure of the data rather than simplifying them as is commonly done, comparative spectral decompositions, such as the GSVD and tensor GSVD, underlie a mathematically universal description of the relationships between a primary tumor's genotype and a patient's overall survival phenotype, which other methods miss.

### Ethics approval

Ethics approval was not required to perform this research.

## SUPPLEMENTARY MATERIAL

See the supplementary material for the Methods section, Figs. S1–S14, and Tables S1–S7, and Datasets S1–S5, which are also available at https://alterlab.org/adenocarcinomas_genotype-phenotype/.
